# Unveiling the mechanical performance of partially replaced coronal restorations in root canal-treated teeth: an in-vitro study

**DOI:** 10.1186/s12903-024-05180-y

**Published:** 2024-12-27

**Authors:** Mohammed Turky, Gianluca Plotino, Nermin Alsayed Mahmoud

**Affiliations:** 1 Department of Endodontics, Faculty of Dentistry, Minia University, Minia, Egypt; 2 Department of Endodontics, Faculty of Dentistry, Sphinx University, Assiut, Egypt; 3 Private Practice, Rome, Italy; 4 Department of Conservative Dentistry, Faculty of Dentistry, Minia University, Minia, Egypt

**Keywords:** Compression fracture, Dental restoration failure, Endodontics, Mechanical stress, Premolar

## Abstract

**Objectives:**

To compare the mechanical performance of partially replaced (repaired) intra-coronal restorations to totally replaced ones in root canal-treated teeth.

**Methods:**

Thirty maxillary second premolars were selected according to strict criteria, mounted on moulds, and had mesio-occluso-distal (MOD) cavities prepared. Resin composite restorative material was used to perform the initial restoration, followed by aging procedures using thermo-mechanical cycling fatigue to replicate six months of intraoral aging. The specimens were then randomly divided into two groups: a totally replaced restoration (TR) group (*n* = 15), which involved the preparation of a traditional endodontic access cavity after the complete removal of the pre-existing coronal filling; and a partially replaced restoration (PR) group (*n* = 15), which involved accessing the tooth through the pre-existing restoration without completely removing it. Root canal preparation and filling procedures were conducted, and the access cavity was sealed with a new resin composite restoration, followed by a new thermo-mechanical cycling aging procedure. Finally, the specimens were submitted to a static fracture test to measure specimen fracture strength and determine the failure mode pattern (repairable fracture or irreparable fracture). Chi-square and *t*-tests were used for statistical analysis.

**Results:**

Significant differences between the groups regarding their mechanical resistance were found. The average failure load of the TR group was 1115.13 N and 1330.23 N in the PR group (*p* = 0.002). Regarding the failure modes, the TR group exhibited eight irreparable fractures, while the PR group had four (*p* = 0.136).

**Conclusions:**

Partially replaced restorations presented higher fracture strength and led to fewer irreparable fractures when compared to totally replaced restorations in root canal-treated teeth.

## Introduction

Endodontic failure might be attributed to either structural or biological factors [1]. Although biological causes of failure, such as the development of new, persistent, or recurrent post-treatment diseases due to microbial factors, represent a major cause of endodontic failure [2], they may provide an opportunity for the preservation of the tooth [3, 4]. On the other hand, structural factors such as restorative failure are one of the most prominent reasons that may threaten the tooth’s survival, and in turn, may result in the loss of root canal-treated teeth [5]. Moreover, numerous studies have emphasized the role that the quality of coronal restoration plays in the success of root canal-treated teeth [6–10]. Thus, the selection and quality of the coronal restoration play a pivotal role in the retention, longevity, and success of root canal-filled teeth [8–10].

As there is no definite standard for the restoration of root canal-treated teeth, it remains one of the challenging issues clinicians need to face in their daily practice [11], and to date, research has not ceased in an attempt to reach a meaningful conclusion regarding this topic [1]. Moreover, the optimal final restoration of root canal-treated teeth must fulfil several requirements such as restoration of the tooth’s function, anatomy, aesthetics, occlusal and proximal contacts, protection and support of the remaining tooth structure, and prevention of coronal leakage [11]. All these requirements should be met whether the tooth has been restored with direct or indirect restorations [12].

Although its limited accessibility and visibility may pose challenges, access cavity preparation through a pre-existing, well-fitted, and adapted indirect restoration may be indicated in some instances for mechanical and financial reasons, with suggestions that it can achieve predictable treatment outcomes [13–15]. However, scientific literature has emphasized the necessity for complete removal of the direct intra-coronal restoration prior to gaining access to the pulp chamber in order to detect any hidden cracks, fractures, and recurrent caries behind the pre-existing restoration [16]. Moreover, this step aims to avoid forcing any restorative fragments into root canals, which may jeopardize further treatment and negatively impact the adaptability of the restoration during drilling, potentially leading to coronal leakage and treatment failure [16].

With the advent of the minimally invasive concept, different access cavity designs have evolved in an attempt to preserve tooth structure [17]. It has been reported that any tooth loss during access cavity preparation would reduce the tooth’s fracture resistance [18]. Total replacement of a pre-existing direct intra-coronal restoration is considered excessively interventional when the restoration is clinically and radiographically intact, and there is no evidence of cracks, fractures, or recurrent caries, as it may involve additional removal of sound tooth structure, subsequently weakening the remaining tooth structure [19, 20]. Moreover, polymerization shrinkage stresses during placement of resin composite restorations may result in fracture of the adjacent tooth structure [20]. Thus, access cavity preparation through a pre-existing coronal restoration may be a minimally invasive approach to minimize excessive tooth loss. This raises the question: could the mechanical performance of the partially replaced coronal filling be equivalent to that of a completely replaced restoration? Given the lack of scientific evidence capable of clarifying this doubt, the present study was designed to respond to and fill this gap in knowledge.

The null hypothesis to be tested was that there would be no differences in the mechanical performance between a partially replaced coronal filling and a totally newly replaced restoration in root canal-treated teeth.

## Materials and methods

### Ethical regulation

The current study was conducted after obtaining approval from the Research Ethics Committee of the Faculty of Dentistry at Minia University, Egypt (Committee No. 103, registration No. 893, date: 30/1/2024).

### Sample size calculation

A power analysis was conducted to ensure adequate power for applying a two-sided statistical test of the null hypothesis, which posits that no difference exists between the tested groups regarding measured fracture resistance. By adopting an alpha (α) level of 0.05, a beta (β) level of 0.2 (power = 80%), and effect size (d) of 1.10, calculated based on the results of a pilot study involving ten samples in each group, the total required sample size (n) was determined to be 30 samples (15 samples per group). Sample size calculation was performed using R statistical analysis software, version 4.3.2 for Windows (Vienna, Austria).

### Sample selection (strict criteria were applied for the selection of the teeth)

A total of 30 freshly and atraumatically extracted human intact mature maxillary second premolars, possessing Vertucci’s type I root canal configuration [21] and nearly straight roots with an equivalent curvature angle of less than 10º as determined by Schneider’s method [22], were collected from the outpatient clinic at the Faculty of Dentistry, Minia University, Egypt after obtaining the respective patient’s informed consent. Anatomical matching for the selected teeth and their pulp morphologies and volumes was achieved using 3D imaging (Papaya 3D plus, Genoray, Gyeonggi-do, Korea). Tooth dimensions were measured using a digital calliper with an accuracy of 0.01 mm. The buccolingual dimension of the selected teeth was 9.5 ± 0.5 mm, while the mesiodistal dimension was 7.5 ± 0.5 mm. To standardize the tooth length for all samples, occlusal reduction was performed, setting the tooth length at 21 mm from the buccal cusp tip as a reference point. Teeth presenting coronal or root caries, calcified canals, pre-existing root canal fillings, teeth extracted more than 1 month before conducting the experiments, and external or internal resorptive lesions were excluded. Additionally, teeth were inspected for evidence of cracks or fractures using a dental operating microscope (DOM) (Magna Labomed, Labo America Inc., 920 Auburn Court Fremont, CA 94538, USA) at a magnification of 20X. Subsequently, the teeth were cleaned from any hard or soft tissue deposits, disinfected for 30 min in a 5.25% sodium hypochlorite solution (NaOCl) (Omez, Phar Omez, Pharaonic Pharmaceuticals, Egypt), and finally immersed in a 0.1% thymol solution (Formula e Acao, São Paulo, SP, Brazil) for the same period until use [23].

### Teeth mounting

**Fig. 1:**
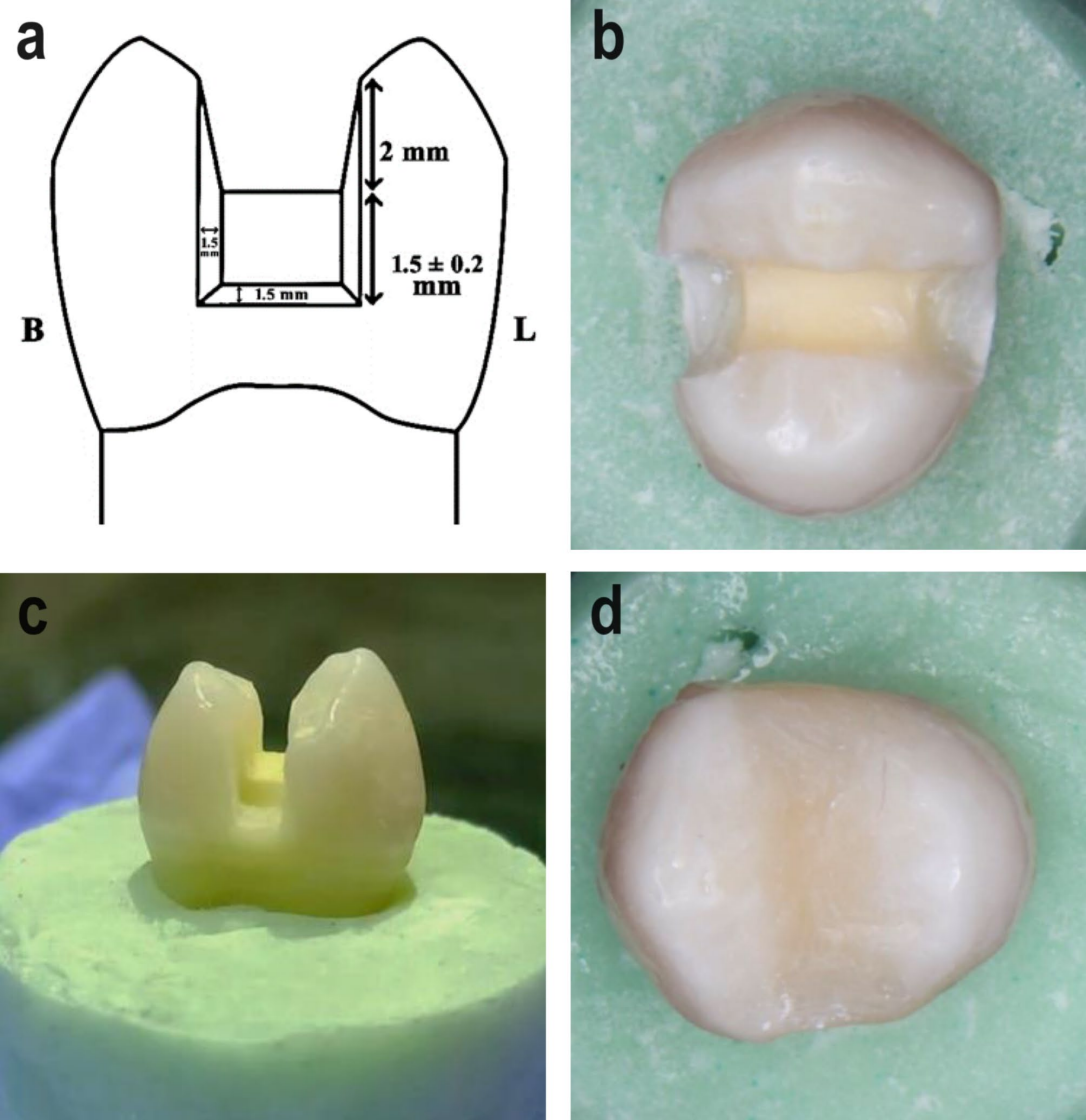
Cavity preparation and initial restorative procedures: (**a**) scheme showing the dimensions of the prepared cavity; (**b**) occlusal view; (**c**) proximal view, (**d**) MOD composite resin initial restoration

Two half-split Teflon moulds, each measuring 25 mm in height and 30 mm in diameter, were utilized. After pouring self-curing resin into the mould, each tooth, with a stretch film surrounding its root, was submerged in acrylic resin up to 2 mm apical to the cementoenamel junction (CEJ) to simulate the bone level. A Dental Surveyor (Ney Dental Surveyor, Anaheim, CA, USA) was employed to position each tooth inside an acrylic block, ensuring proper centering and alignment parallel to the tooth’s long axis. The teeth with the stretch film were taken out once the acrylic resin had hardened and reinserted after a light body silicone (Elite HD; Zhermack SpA, Badia Polesine, Italy) was injected into the root cavity to mimic the periodontal ligament. Any extra impression material was removed using a #12 scalpel. Mesio-occluso-distal (MOD) cavities were prepared using a carbide bur (straight round end fissure #245; Kerr, Kloten, Switzerland) operated in a high-speed handpiece (Sirona, Erlangen, Germany) with water cooling. The occlusal isthmus width was set at one-third of the intercuspal distance, with measurements of 2 ± 0.2 mm pulpal depth, 1.5 ± 0.2 mm axial height, 1.5 ± 0.2 mm axial depth, and 1.5 ± 0.2 mm gingival width (Fig. 1a-c) [24].

### Restorative procedures

All restorative procedures were carried out by a single specialist with 23 years of experience in Restorative Dentistry (N.E.M.). The enamel cavity walls of each prepared premolar were etched using 37% phosphoric acid etching gel (Total etch, Ivoclar Vivadent, Zurich, Switzerland), applied for 15 s, rinsed with water for an additional 15 s, and gently air-dried. Per the manufacturer’s instructions, after evenly applying the bonding agent (Adhese universal, Ivoclar Vivadent) to the cavity walls, the material was rubbed for 20 s with a micro brush (3MTM XS Applicator Brushes; 3 M St. Paul, MN, USA), then gently dried with oil-free air to remove any remaining solvents. Subsequently, the material was light-cured for 20 s using a light-emitting diode curing unit (3 M Elipar Deep Cure-S LED Curing Light; 3 M St. Paul, MN, USA) with a light intensity of 1470 mW/cm². Each tooth was encircled by a Tofflemire matrix system (Medicaline, Bunkyo-ku, Japan) before composite packing, and a gold-plated composite applicator (American Eagle composite set, West Chester Township, Ohio, United States) was utilized to pack the resin composite restorative material A3 (Tetric N-Ceram, Ivoclar Vivadent, Zurich, Switzerland) incrementally into each prepared cavity using the oblique layering technique in order to reduce the c-factor and prevent distortion of the cavity walls [25–28]. Following the manufacturer’s instructions, each increment was light-cured for 20 s. To achieve the proper mesiodistal and occlusal contour, as well as the ridges and inclines of the occlusal anatomy, the last 2 mm increment was placed occlusally to over-fill the cavity, then light-cured for 20 s. Finishing and polishing were conducted using the TOR VM Finishing and Polishing Kit (TOR VM, Moscow, Russia) (Fig. 1d).

### Aging procedures (thermo-mechanical cycling fatigue)

A four-station multi-modal ROBOTA chewing simulator (ACTA Fatigue tester, Amsterdam, Netherlands) with servomotor control (Model Ach-09075dc-T, Ad-Tech Technology, Berlin, Germany) and a thermo-cyclic protocol was employed to conduct the mechanical aging test. Using ROBOTA, simultaneous motions in both vertical and horizontal directions under thermodynamic conditions were simulated. Each chamber consists of an upper, hardened steel stylus holder that can be securely screwed tight to serve as an antagonistic material and a lower plastic sample holder where the specimen can be embedded. Five kilograms of weight, equivalent to 49 N of chewing force, were applied. The following settings were employed for the chewing simulation: a cycle frequency of 1.6 Hz, rising/forward speed of 90 mm/s, descending/backward speed of 40 mm/s, horizontal movement of 1 mm, and rising/vertical movement of 3 mm. The specimens underwent 75,000 cycles with 6000 thermal cycles (5˚/55˚C, dwell period of 25 s) to replicate six months of intraoral aging [29].

### Specimen grouping

Teeth were numbered and randomly allocated into two groups based on whether the coronal restoration was entirely or partially replaced: the totally replaced restoration (TR) group (*n* = 15) involved the preparation of a traditional access cavity after the complete removal of the pre-existing coronal restoration, while the partially replaced restoration (PR) group (*n* = 15) involved accessing the tooth through the pre-existing restoration without the need for its complete removal. All endodontic and subsequent restorative procedures were performed by a single endodontist with 20 years of experience in endodontics (M.T.).

**Fig. 2:**
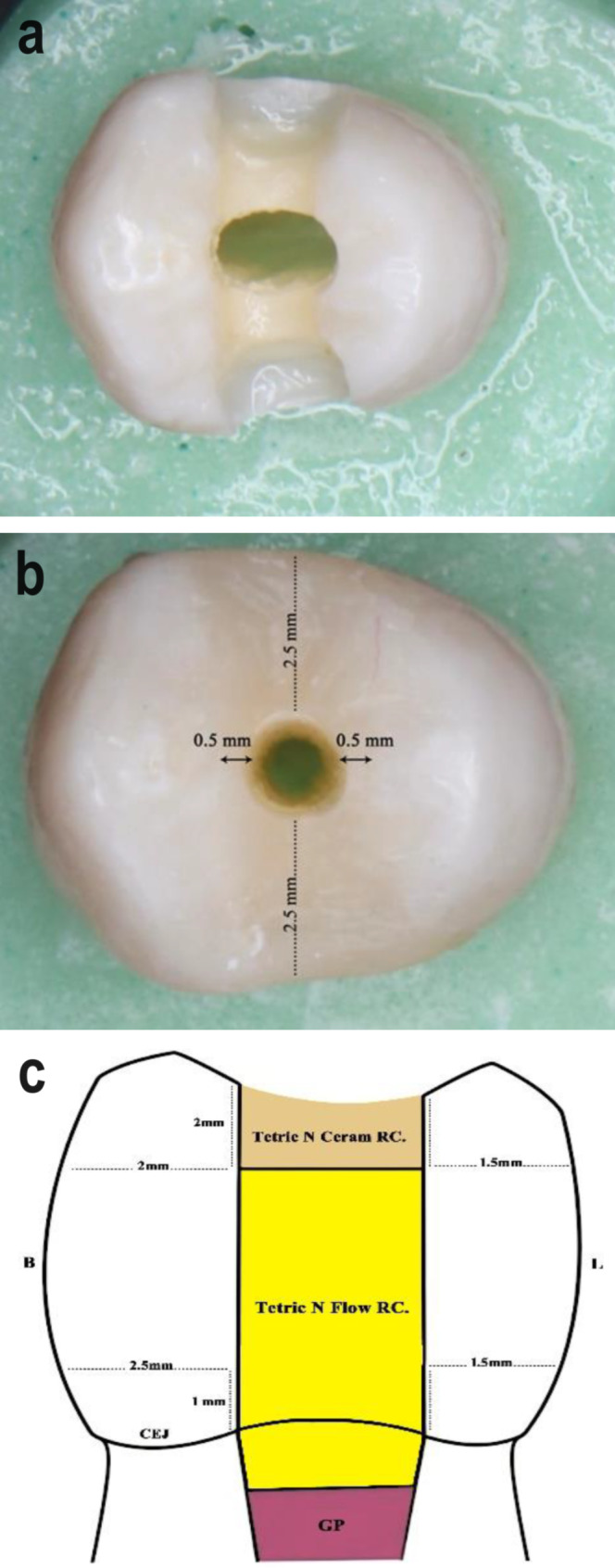
Gaining endodontics access: (**a**) totally replaced restoration access; (**b**) partially replaced restoration access; (**c**) illustration of the post-endodontic restoration

TR group: After completely removing the pre-existing coronal restoration, a traditional access cavity was prepared following common guidelines for access cavity preparation, aiming to establish straight-line access to canal orifices with smoothly divergent cavity walls [30, 31]. This design was achieved using a round diamond bur #801 − 014 (Komet, Brasseler, Lemgo, Germany) to remove the entire roof of the pulp chamber. Subsequently, the cavity walls were refined using a tapered carbide fissure bur (Komet H33L, Komet Brasseler). All access cavity burs were mounted on a high-speed handpiece with water spray. The access cavities were prepared while maintaining the following wall thicknesses, as outlined in an earlier study by Garlapati et al. [32]: the buccal wall measured 2 mm at the occlusal surface and 2.5 mm at the gingival floor level, and the palatal wall measured 1.5 mm at both gingival floor level and occlusal surface. The widths of the remaining wall thickness were measured using a digital calliper (Fig. 2a).

PR group: Under a magnification of 16X using a dental operating microscope (DOM), an access cavity was prepared through the pre-existing restoration using a diamond round bur # 806 − 010 (Komet, Brasseler), mounted on a high-speed handpiece with water spray, with maximal preservation of the remaining restoration and tooth structure. The access opening was standardized in all samples, leaving 0.5 mm of the restoration buccally and palatally, while 2.5 mm of the restoration was left mesial and distal to the access cavity (Fig. 2b).

### Root canal preparation and filling

Root canal preparation and filling procedures were similar for both groups. After access cavity preparation, patency was verified using a manual stainless-steel K-file ISO size 10 (Dentsply, Maillefer, Ballaigues, Switzerland). Any teeth failing to demonstrate patency were excluded from the study. Working length was visually determined by inserting a stainless-steel K-file ISO size 10 into the root canal until it became visible through the apical foramen, then subtracting 1 mm from this measurement. All teeth were instrumented up to size 40/0.04 (Hyflex CM, Coltene Whaledent, Cuyahoga Falls, OH, USA) and received identical volumes of irrigation according to the standard protocol, which involved using 2 ml of 5.25% NaOCl solution for 20 s between files, followed by a final rinse alternating between 10 ml of 5.25% NaOCl solution for 2 min and 10 ml of 17% ethylenediaminetetraacetic acid (EDTA) solution for 2 min, with an intermediate rinse using the same volumes of saline solution [33, 34]. A final flush with saline solution was performed to eliminate any remaining irrigant residues [35]. After root canal preparation was completed, the canals were dried using size 40/0.04 paper points (Coltene Whaledent) and obturated using a single-cone technique with a matched master gutta-percha cone size 40/0.04 (Coltene Whaledent) and a bioceramic sealer (EndoSequence BC sealer, Brasseler Blvd, Savannah, USA). The coronal end of the root canal filling was standardized to be 1 mm below the canal orifices. The pulp chamber was cleaned using a cotton pellet soaked in 70% alcohol to remove any sealer residues. The quality of obturation was assessed using conventional periapical radiographs.

### Final coronal restoration

TR group: Upon completion of endodontic procedures, the access cavity was sealed with a new resin composite restoration. This involved etching the cavity enamel surfaces, applying adhesive to all cavity walls, and using bulk-fill flowable resin composite (Tetric N-Flow Bulk Fill, Ivoclar Vivadent, Zurich, Switzerland ) according to the manufacturer’s instructions to fill most of the cavity, leaving only 2 mm for the final resin composite filling (Tetric N-Ceram, Ivoclar Vivadent, Zurich, Switzerland). The restoration was then packed, cured, finished, and polished as previously described (Fig. 2c).

PR group: Acid etching and bonding application were carried out as previously described for the surfaces of the access cavity and old composite restoration. Bulk-fill flowable resin composite (Tetric N-Flow Bulk Fill, Ivoclar Vivadent, Zurich, Switzerland ) was applied to fill most of the access cavity and light-cured for 20 s, leaving only 2 mm for the final resin composite filling (Tetric N-Ceram, Ivoclar Vivadent, Zurich, Switzerland ). This was followed by light curing for 20 s, finishing, and polishing (Fig. 2c).

### Final aging procedures (thermo-mechanical cycling fatigue)

The final coronal restoration was aged using the Robota chewing simulator for an additional 75,000 cycles with 6000 thermal cycles (5˚/55˚C, dwell period of 25 s), equivalent to six months of intraoral aging.

### Measurement of fracture resistance (static fracture test)

All samples were individually mounted on a computer-controlled materials testing machine (Model 3345; Instron Industrial Products, Norwood, MA, USA) equipped with a load cell of 5 kN, and data were recorded using computer software (Bluehill Lite Software, Instron^®^). The samples were secured to the lower fixed compartment of the testing machine by tightening screws. The fracture test was conducted by applying compressive load occlusally in a direction parallel to the long axis of the root using a metallic rod with a round tip (2.6 mm diameter) attached to the upper movable compartment of the testing machine, which travelled at a cross-head speed of 1 mm/min. The load at failure was indicated by an audible crack and confirmed by a sharp drop in the load-deflection curve recorded using computer software (Bluehill Lite Software, Instron^®^ Instruments). The load required to cause fracture was recorded in Newton.

### Failure mode assessment

Failure modes were examined by an independent evaluator who was blinded to the experimental procedures using a USB digital microscope (U500x Digital Microscope, Guangdong, China) at a magnification of 35X. Images were captured and transferred to a personal computer equipped with image analysis software (Image J 1.43U, National Institute of Health, USA) to determine the failure mode pattern, whether it was a repairable fracture (where the restoration’s fracture would occur above the CEJ) or an irreparable fracture (where the fracture of the coronal restoration would occur below the CEJ) [36].

The images were captured using the following image acquisition system: a digital camera (U500x Digital Microscope, Guangdong, China) with 3 megapixels of resolution, positioned vertically at a distance of 2.5 cm from the samples; the angle between the axis of the lens and the sources of illumination was approximately 90°; plus illumination was provided by 8 LED lamps (adjustable by the control wheel), with a colour index close to 95%. The images were captured at maximum resolution and connected to a compatible personal computer using a fixed magnification of 35X. Each image was recorded with a resolution of 1280 × 1024 pixels.

### Statistical analysis

The Shapiro-Wilk test was used to assess the assumption of normality of the fracture resistance test outcomes. Considering the Gaussian distribution of the outcomes, the groups with totally or partially replaced restorations, whose results were expressed in means and standard deviations, were compared using the Independent Samples *t*-test. Regarding the results of failure modes, the Chi-square test was used to compare the types of failure modes across the analyzed groups. The significance level was set at 5% (SPSS v22.0 for Windows; SPSS Inc., IBM, Zurich, Switzerland).

## Results

The results of the fracture resistance test showed a mean failure load of 1115.13 ± 200.76 N in the group with totally replaced restorations and 1330.23 ± 161.89 N in the group with partially replaced restorations. There was a statistically significant difference between the groups (*p* = 0.002) (Table 1).

**Fig. 3:**
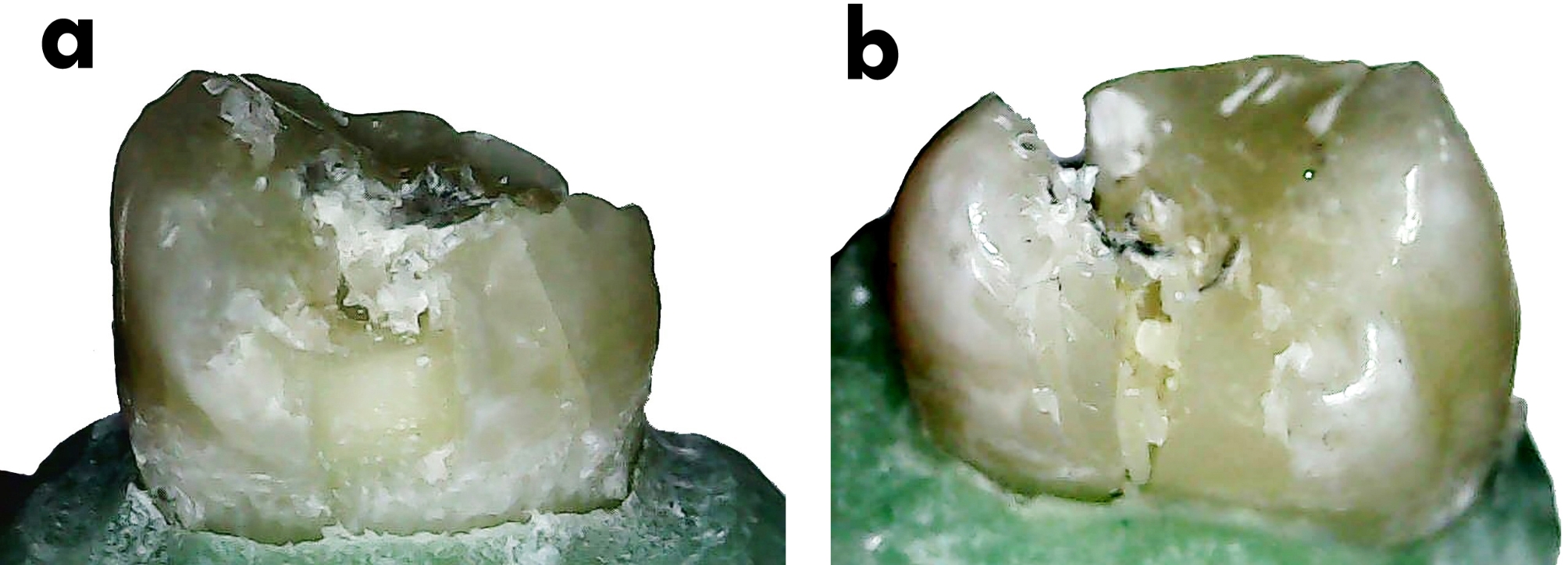
Representative images of the failure mode assessment: (**a**) repairable failure; (**b**) irreparable failure

In terms of failure modes, the group with totally replaced restorations had 8 irreparable fractures, while the group with partially replaced restorations had 4. There was no statistically significant difference between the groups (*p* = 0.136) (Fig. 3a-b) (Table 1).

**Table 1:** Fracture test and failure mode assessment results

Group	*n*	Fracture test	Failure mode	
		(Newtons)	Repairable	Non-Repairable
Totally replaced restorations	15	1115.13 ± 200.76^a^	7	8
Partially replaced restoration	15	1330.23 ± 161.89^b^	11	4

Different superscript letters represent statistically significant differences within that column (*P* < 0.05)

## Discussion

While a substantial body of literature attributes most endodontic failures to factors causing the emergence or persistence of endodontic diseases [2, 37, 38], restorative complications stand out as one of the most common reasons for the extraction of root canal-treated teeth [39, 40]. This has led to increased attention to the importance of coronal restoration [41]. Indeed, it is evident that the coronal restoration of root canal-treated teeth significantly influences long-term treatment outcomes, including success rates and the survival of treated teeth [5, 9, 10, 42]. However, selecting the most appropriate restoration remains one of the most challenging decisions, largely dependent on each individual case [11].

Traditional access cavities typically involve the removal of unsupported tooth structures, caries, and, if present, pre-existing restorations [30]. The primary factors influencing the fracture resistance of root canal-treated teeth are the amount and quality of the remaining tooth structure [43]. Establishing access to the canal orifices through a pre-existing coronal restoration may allow for more conservation of tooth structure. However, there is currently insufficient data in endodontic literature to determine whether a partially replaced direct restoration exhibits superior or inferior mechanical behaviour compared to a completely replaced one. Therefore, the present study aimed to compare the biomechanical behaviour of a partially replaced restoration to that of a completely replaced restoration in root canal-treated teeth.

Maxillary premolars are particularly susceptible to fracture due to their small crown volumes and unfavourable crown-to-root proportions [44, 45]. Research has demonstrated that even non-functional cusps of maxillary premolars are prone to fracture [46]. It is notable that MOD cavities in root canal-treated teeth, especially in maxillary premolars, have been reported to increase their susceptibility to fracture [47, 48]. Given these circumstances, meticulous selection of the most suitable coronal restoration is necessary to reinforce the remaining tooth structure. Although using fiber posts has been suggested to restore root canal-treated teeth, particularly maxillary premolars, with MOD cavities [49–51], numerous publications have supported the use of direct resin composite restorations without the need for any post, stressing the concept of preserving the fracture resistance of treated teeth [1, 52–55]. Apart from its role in preserving tooth structure and offering superior aesthetic qualities, resin composite restoration has been shown to internally reinforce root canal-treated teeth, even without cuspal coverage [56–59]. Therefore, in the current study, MOD cavities in root canal-treated maxillary single-rooted second premolars were restored with direct intra-coronal resin composite.

In an effort to address the significant disparity observed in most fracture test investigations [60], sample selection and preparation in the present study were conducted according to strict criteria. Freshly and atraumatically extracted intact teeth, devoid of any evidence of cracks and fractures, were chosen, ensuring consistency in tooth type, dimensions, morphology, pulp space volume, and morphology through 3D anatomical matching. These teeth were then stored in a suitable preservative medium for a short duration until use. Additionally, cavity preparation during restorative and endodontic procedures adhered to identical dimensions, and the root canal procedures were standardized. The selected teeth underwent thermo-mechanical fatigue (aging) to simulate conditions encountered in the oral environment and enhance the reliability of the results, as previous research has highlighted the significant impact of tooth/restoration age on fracture strength [61, 62].

Mechanical aging involves repeated mechanical forces that create fluctuating stresses in the restorative material and adjacent tooth, leading to fatigue degradation and potential failure from accumulated minor or major damage [63]. Mechanical loading may be applied associated with simultaneous thermal cycling or the thermal cycling can be performed as a separate step prior to loading. Thermal cycling is a method used to assess the durability of restorations by subjecting them to expansion and contraction stresses through alternating immersion in liquids at different temperatures. These temperature changes can strain the composite material, restored structures, and their bonded interfaces. The various components within the composite, such as the polymer matrix and filler particles, have different thermal expansion rates, leading to stress in the bonds between them and with the tooth structure [63]. It has been reported that a simultaneous combination of mechanical and thermal cycling can generate faster mechanical degradation and fatigue of resin composite restorations [63].

There are two methods for testing fracture resistance: dynamic and static testing. Since the static fracture test is the most widely used method due to its effectiveness and capacity to yield comparable results, it was utilized in this investigation [64]. Although dynamic testing would replicate the clinical setting, it has been demonstrated that variations in its design can impair the correlation of results [64]. Therefore, this latest method was not considered presently.

The findings of the current study appear to be able to reject the null hypothesis tested, indicating that the partially replaced restoration exhibits statistically significant higher fracture resistance (*p* = 0.002) and a tendency to present a failure mode with more reparable fractures (*p* = 0.136) when compared to the completely replaced restoration. This might be attributed to the retention of the majority of restoration parts in the partially replaced restoration, which prevents the introduction of new elements that could compromise bonding to the tooth structure [65]. Moreover, complete replacement of the restoration is typically associated with unnecessary removal of the surrounding tooth structure, resulting in subsequent weakening of the supporting structure and a larger restoration that performs less effectively than a smaller one [20]. It has been noted that total restoration replacement accelerates the restoration death spiral [20].

The present findings suggest that restoration repair is preferred over complete restoration replacement as long as there are no signs of recurrent caries, marginal discrepancies, or evidence of cracks or fractures. This approach helps preserve more tooth structure and saves time and cost.

One of the shortcomings of the present study is the use of the same type and brand of resin composite restoration to repair the coronal restoration in the PR group. Although better, this does not reflect the actual situation in daily clinical practice. Endodontic treatment may be performed by a different practitioner than the one who made the pre-existing restoration, and details of the previous restoration may not be recorded in the patient’s file. Additionally, the same previous restoration may not be commercially available. Therefore, further studies are required using a different type and brand of resin composite from the pre-existing one. Another limitation of the current investigation is that it was conducted in vitro, making it impossible to test the fracture resistance of subjects clinically. Despite its in vitro nature, this study attempted to consider most factors that might increase the risk of bias in the fracture test. To enhance the validity of the present results, future long-term survival studies are needed to compare the durability of the repaired restoration to the completely replaced one in clinical settings. Moreover, access preparation through a pre-existing restoration should be investigated regarding its influence on subsequent endodontic procedures and the fracture resistance of the treated teeth. As the present study was limited to evaluating the mechanical performance of the repaired restoration itself compared to the completely replaced one, further studies are required to assess the fracture resistance of the root canal-treated teeth restored with such a minimally invasive approach.

## Conclusions

Within the limitations of this in vitro investigation, it was possible to conclude that the repaired restoration was superior to the completely replaced one from a mechanical perspective. Gaining access to the pulp chamber through a direct intra-coronal restoration provides a viable alternative to the traditional concept that involves removing the entire restoration.

## Data Availability

Availability of the data and materials: All data or materials generated or analyzed during this study are included in this article.

## Declarations

### Clinical trial number

Not applicable. Conducting the current experiment was limited to obtaining approval from the local Research Ethics Committee at the Faculty of Dentistry, Minia University, Egypt (Committee No. 103, registration No. 893, date: 30/1/2024).

## Abbreviations

3D: Three-dimensional
CEJ: Cementoenamel junction
DOM: Dental operating microscope
EDTA: Ethylenediaminetetraacetic acid
HZ: Hertz
ISO: International standard organization
LED: Light emitting diode
mm/min: Milemeter per minutes
mm/s: Milemeter per seconds
MOD: Mesio-occluso-distal
N: Newten
NaOCl: Sodium hypochlorite
PR: Partially replaced
TR: Totally replaced
